# Predictive performance of critical illness scores and procalcitonin in sepsis caused by different gram-stain bacteria

**DOI:** 10.6061/clinics/2021/e2610

**Published:** 2021-05-27

**Authors:** ShengTao Yan, GuoQiang Zhang

**Affiliations:** Department of Emergency Medicine, China-Japan Friendship Hospital, Chaoyang District, Beijing 100029, P.R China.

**Keywords:** Sepsis, Shock, Septic, Procalcitonin, Prognosis

## Abstract

**OBJECTIVES:**

To compare the early and late predictive values of several critical illness scores (CISs) and biomarkers in sepsis-3 patients with bloodstream infections (BSIs) and to identify the prognostic value of procalcitonin (PCT) for different gram-stain bacteria infections.

**METHODS:**

Patients with at least one positive blood culture within 24h of emergency department admission and with a final diagnosis of sepsis/septic shock were enrolled. CISs were calculated based on the first parameters on the day of admission. The receiver operating characteristics curve was used to analyze the predictive value of CISs and biomarkers for early and late mortality.

**RESULTS:**

Of 834 enrolled patients with sepsis-3, death occurred in 214 patients within 28 days and in 273 patients within 60 days. Compared with biomarkers, CISs showed a significantly higher area under the curve (AUC) in the prediction of early and late mortality (*p*<0.01), especially for patients with GNB infection. The Sequential Organ Failure Assessment score showed a higher AUC for predicting early mortality than the Mortality in Emergency Department Sepsis score (*p*=0.036). Compared with GNB infections, the AUC values of the PCT for gram-positive bacteria (GPB) infections were higher for predicting early or late mortality; PCT showed higher AUC than high-sensitivity C-reactive protein and white blood cells for predicting early mortality (*p*<0.05).

**CONCLUSIONS:**

CISs were more advantageous in the assessment of early and late prognosis, especially for patients with GNB infections; however, for sepsis with GPB infection, PCT can be used for the prediction of early mortality.

## INTRODUCTION

Sepsis is one of the leading causes of critical illness and mortality worldwide (1-3) and has been increasing annually by 8%-13% over the past decade, primarily due to the increase in invasive procedures, immunosuppressive drugs, chemotherapy, and transplantation (4). The emergency department (ED), the main healthcare segment treating critically ill patients, requires effective strategies to identify patients at high risk for death, either through a readily available laboratory parameter or a clinical decision rule that can be assessed rapidly. However, it has been proven difficult to develop clinical and laboratory criteria that accurately predict the risk for mortality. Several potential scores for timely diagnosis, risk stratification, and evaluation of the prognosis of sepsis in the ED and intensive care unit (ICU) have come into focus in the last decade (5-8). The Mortality in Emergency Department Sepsis (MEDS) score (Supplemental File 1) (9) incorporates available information in the ED to aid clinicians in accurately assessing a patient’s disease severity and mortality risk for ED presentation (10,11). The Sequential Organ Failure Assessment (SOFA) score (Supplemental File 2) (12) uses clinical and laboratory variables to rank the severity of organ failure and is associated with mortality in ED patients hospitalized with sepsis (13,14). Acute Physiology and Chronic Health Evaluation II (APACHE-II) is now the most widely used severity-of-illness score worldwide; it is used to predict the mortality rate of critically ill patients admitted to the ICU. The logistic organ dysfunction system (LODS) score (Supplemental File 3) (15) has also been proven to be valid for predicting ICU mortality in critically ill patients (6,16).

Sepsis management guidelines recognize the value of biomarkers in providing supplemental data that can support clinical assessment and treatment decisions (17). A few nonspecific biomarkers are established markers for the prognostic evaluation of patients with sepsis. Procalcitonin (PCP), a peptide precursor of the hormone, calcitonin, has been shown to be an important reference marker for infection (18,19). C-reactive protein (CRP) is an acute-phase reactive protein that can interact with capsule C polysaccharides of *Streptococcus pneumoniae*. Previous studies have shown that PCT and CRP concentrations are related to the prognosis of patients with sepsis (20,21). The use of such parameters may improve the accuracy of infection prognosis.

Given that few studies have compared the various outcome-prediction scoring systems and biomarkers for sepsis and septic shock patients according to the sepsis-3 criteria established by the Society of Critical Care Medicine/European Society of Intensive Care Medicine task force (22), the objective of this work was to identify the critical illness scores (CISs) and biomarkers that better predict sepsis-related mortality by comparing predictive validity for early (28-day) and late (29- to 60-day) mortality among patients with bloodstream infections (BSIs) in the ED. Further analysis was performed to identify the prognostic value of PCT for different gram-stain bacteria infections.

## SUBJECTS AND METHODS

This retrospective observational study evaluated patients with BSIs admitted to the ED of a teaching hospital at Peking University Medical School with 200,000 ED visits per year between January 2014 and December 2018. The study was approved by the China-Japan Friendship Hospital Ethics Committee (2017-110).

### Data source

We reviewed the medical records of patients aged >18 years who had visited our ED with infection-related disease and who underwent at least one positive blood culture and laboratory tests, including PCT and high-sensitivity CRP (hs-CRP), within 24h of ED arrival. For patients with a mean arterial blood pressure (MAP) <65 mmHg or lactate concentration ≥2 mmol/L on ED admission, MAP and lactate concentration after fluid resuscitation were also collected to diagnose septic shock. The exclusion criteria were age <18 years, positive blood culture after 24h of ED arrival, repeated positive blood culture at one episode, missing data, contamination, or loss to follow-up.

### Data collection

As previously described (23), patients’ basic information, including age, sex, comorbidities, vital signs, laboratory results, imaging results, vasopressor use, and urine output, was obtained from the database. Only parameters that appeared for the first time after ED arrival were used. SOFA, LODS, MEDS, and APACHE II scores were calculated to identify patients at high risk for poor outcomes from the individual data elements collected within 24h after ED arrival. The primary outcomes were early (28-day) and late (29- to 60-day) all-cause mortality.

### PCT levels and blood cultures

Plasma PCT assessment is usually performed in the ED in patients with clinically suspected sepsis. As previously described (23), serum PCT levels were measured using an automatic analyzer (Vidas B.R.A.H.M.S.; bioMérieux, Marcy l’Etoile, France), according to the manufacturer’s instructions. The lower detection limit of the assay was 0.05 ng/mL, and the assay sensitivity was 0.09 ng/mL.

According to well-established methods (32), for each sample, an aliquot of 5-10 mL whole blood was inoculated into Bactec™ aerobic and anaerobic bottles (Becton Dickinson, Sparks, MD, USA) that were then incubated in a Bactec™ FX automated blood culture system (Becton Dickinson). Aliquots were removed from positive cultures for Gram staining and streaked on a solid medium for subsequent analysis. Microorganisms were identified using conventional methods and matrix-assisted laser desorption/ionization-time-of-flight mass spectrometry (Bruker Daltonik, Bremen, Germany).

### Pathogen identification

As previously described (23), microorganisms detected in blood cultures (BCs) are considered clinically relevant pathogens rather than contaminants if they meet the following conditions: (1) detection of two or more BCs and reported by the clinician as the cause of the infection episode; (2) detection in one set of BCs but consistent with the results of cultured samples from suspected infectious foci collected from the same patient during the same infectious episode; (3) detection in one set of BCs of a species included among the etiopathogenic agents of the patient’s infectious disease; and (4) detection in one set of BCs reported by the clinician as the cause of the infection episode in the final diagnosis based on clinical, instrumental, and laboratory data. Coagulase-negative staphylococci, *Corynebacterium* spp., and other skin commensals were considered contaminants when isolated from one set of BCs alone (24) and in the absence of clinical and/or laboratory data suggesting a pathogenic role.

### Definition of sepsis and septic shock

Sepsis was defined as life-threatening organ dysfunction due to a dysregulated host response to infection. Sepsis clinical criteria: organ dysfunction is defined as an increase of ≥2 points in the SOFA score. Septic shock was defined as an MAP <65 mmHg and lactate concentration ≥2 mmol/L despite fluid resuscitation with ≥30 mL/kg of crystalloid or 5% albumin in patients diagnosed with sepsis (1).

### Statistical analyses

Statistical analyses were performed using MedCalc version 19.0.4 (MedCalc Software, Ostend, Belgium) and SPSS Version 23.0 software (IBM Corp., Armonk, NY, USA). Quantitative data were presented as means±standard errors of the mean or as the median and interquartile range (i.e., 25^th^-75^th^ percentiles), depending on the distribution of the data. Categorical data are summarized as ratios and percentages. Differences in means for continuous variables were compared using Student’s t-test (two groups) or analysis of variance (multiple groups); differences in proportions were tested using an R×C contingency table and Pearson’s chi-squared or Fisher’s exact test as appropriate. Receiver operating characteristic (ROC) curve analysis and calculation of area under the curve (AUC) were conducted to determine the diagnostic utility of various PCT cut-offs and to assess the ability to predict mortality. Youden’s indices (sensitivity+specificity−1) were calculated to determine the ideal discriminatory cut-off values. All statistical analyses were performed with SPSS 23.0, except for the comparison of AUCs, which was performed using MedCalc. All analyses were exploratory; a two-tailed *p-*value<0.05 was considered as the cut-off for statistical significance.

## RESULTS

### Clinical characteristics

**Figure 1:**
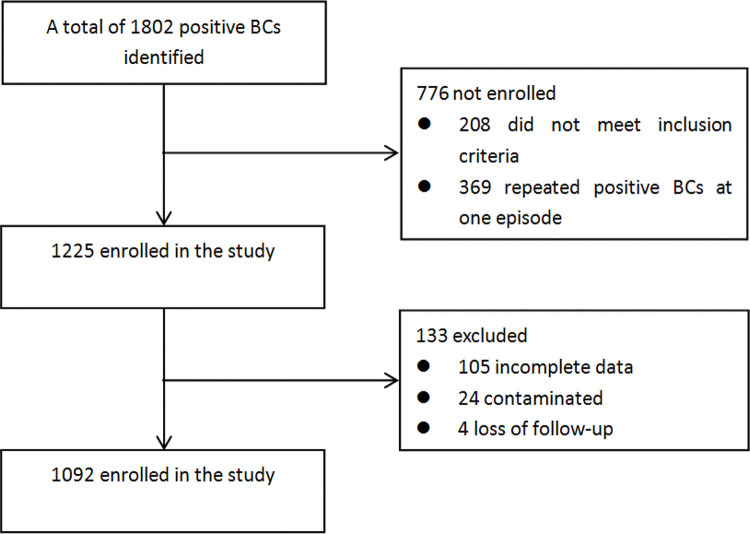
Study enrollment flowchart. BCs, Blood cultures.

A total of 1092 positive BCs were obtained; 834 met the criteria for sepsis-3. A flow diagram of the included and excluded patients is shown in Figure 1. The patients’ demographic and clinical characteristics are shown in Table 1: 522 (62.6%) were men and 312 (37.4%) were women; 666 (79.9%) fulfilled the criteria for sepsis, and 168 (20.1%) fulfilled the criteria for septic shock. Upon final diagnosis, most of the patients were diagnosed with respiratory infections (510 patients, 61.2%), followed by 186 (22.3%) with abdominal infections, 49 (5.9%) with urinary infections, 60 (7.2%) with catheter-related infections, and 10 (1.2%) with infective endocarditis. Regarding the pathogens detected, 393 patients (47.1%) were infected with gram-negative bacteria (GNB), slightly more than those infected by GPB (324 patients, 38.8%). Compared with the sepsis group, PCT and hs-CRP levels, SOFA, MEDS, LODS, and APACHE-II scores, and early and late mortality rates were found to be significantly higher in the septic shock group.

### Comparison of the prediction of early and late mortality according to CISs and biomarkers

**Figure 2:**
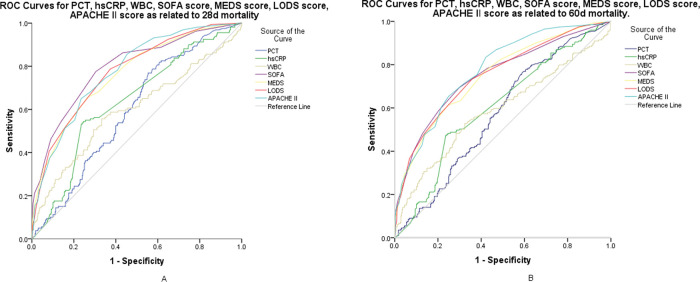
ROC curves for the prognosis of early (A) and late (B) mortality among patients with sepsis and septic shock. A: AUC demonstrates that serum PCT measures 0.634 (95% CI, 0.586-0.682), hs-CRP measures 0.640 (95% CI, 0.587-0.692), WBC measures 0.595 (95% CI, 0.536-0.654), SOFA score measures 0.805 (95% CI, 0.763-0.847), MEDS score measures 0.761 (95% CI, 0.715-0.807), LODS score measures 0.785 (95% CI, 0.742-0.827), and APACHE II score measures 0.778 (95% CI, 0.736-0.821) for the prognosis of early mortality. B: AUC demonstrates that serum PCT measures 0.596 (95% CI, 0.548-0.645), hs-CRP measures 0.605 (95% CI, 0.555-0.656), WBC measures 0.586 (95% CI, 0.532-0.640), SOFA score measures 0.754 (95% CI, 0.709-0.799), MEDS score measures 0.744 (95% CI, 0.699-0.788), LODS score measures 0.755 (95% CI, 0.711-0.799), and APACHE-II score measures 0.774 (95% CI, 0.734-0.815) for prognosis of late mortality. ROC, receiver operating characteristic; AUC, area under the curve; PCT, procalcitonin; CI, confidence interval; hs-CRP, high sensitivity C-reactive protein; WBC, white blood cell; SOFA, Sequential organ failure assessment; LODS, Logistic organ dysfunction system; MEDS, Mortality in emergency department sepsis; APACHE-II, Acute Physiology and Chronic Health Evaluation II.

For the prediction of mortality in the first 28 days, SOFA, MEDS, LODS, and APACHE-II resulted in scores of 8.0 (6.0-11.0), 14.0 (11.0-16.0), 6.0 (4.0-7.0), and 21.0 (16.0-26.0), respectively (*p*<0.001); PCT, hs-CRP, and white blood cells (WBCs) resulted in values of 3.03 ng/mL (0.87-16.09), 117.69 mg/L (89.16-130.00), and 12.42×10^9^/L (6.98-17.35), respectively (*p*<0.01) (shown in Table 2). Figure 2-A shows the ROC curves for the SOFA, LODS, MEDS, and APACHE-II scores and PCT, hs-CRP, and WBC for early mortality. Pairwise comparisons of the AUC of the scores were as follows: SOFA *versus* MEDS, *p*=0.036; SOFA *versus* LODS, *p*=0.213; SOFA *versus* APACHE-II, *p*=0.385; MEDS *versus* LODS, *p*=0.227; MEDS *versus* APACHE-II, *p*=0.134; and LODS *versus* APACHE-II, *p*=0.899. Pairwise comparisons of the AUC of the biomarkers were as follows: PCT *versus* hs-CRP, *p*=0.678; PCT *versus* WBC, *p*=0.239; and hs-CRP *versus* WBC, *p*=0.127. At a cutoff value of 5.0, the SOFA scores showed a sensitivity of 88.5%, a specificity of 56.5%, and a negative predictive value (NPV) of 92.7%; at a cut-off value of 0.84 ng/mL, PCT showed a sensitivity of 88.5%, a specificity of 56.5%, and an NPV of 92.7% (shown in Table 3).

For the prediction of mortality from 29 days up to 60, SOFA, MEDS, LODS, and APACHE-II resulted in scores of 7.0 (5.0-10.0), 13.0 (11.0-16.0), 5.0 (3.0-7.0), and 20.0 (16.0-25.0), respectively (*p*<0.001); PCT, hs-CRP, and WBC resulted in values of 2.75 ng/mL (0.69-13.24), 100.93 mg/L (89.16-126.50), and 11.98×10^9^/L (6.97-16.98), respectively (*p*<0.01) (shown in Table 2). Figure 2-B shows the ROC curves for the SOFA, LODS, MEDS, and APACHE-II scores and PCT, hs-CRP, and WBC for late mortality. Pairwise comparisons of the AUC of the scores were as follows: SOFA *versus* MEDS, *p*=0.374; SOFA *versus* LODS, *p*=0.873; SOFA *versus* APACHE-II, *p*=0.304; MEDS *versus* LODS, *p*=0.458; MEDS *versus* APACHE-II, *p*=0.038; and LODS *versus* APACHE-II, *p*=0.223. Pairwise comparisons of the AUC of the biomarkers were as follows: PCT *versus* hs-CRP, *p*=0.666; PCT *versus* WBC, *p*=0.638; and hs-CRP *versus* WBC, *p*=0.411. At a cut-off value of 5.0, SOFA scores showed a sensitivity of 79.7%, a specificity of 56.8%, and an NPV of 83.8%; at a cut-off value of 0.60 ng/mL, PCT showed a sensitivity of 83.2%, a specificity of 38.8%, and an NPV of 80.6% (Table 3). Compared with the biomarkers, CISs showed a significantly higher AUC for predicting either early or late mortality (*p*<0.01).

### Comparison of the predictive values for early and late mortality according to biomarkers with different gram stain bacteria infection

**Figure 3:**
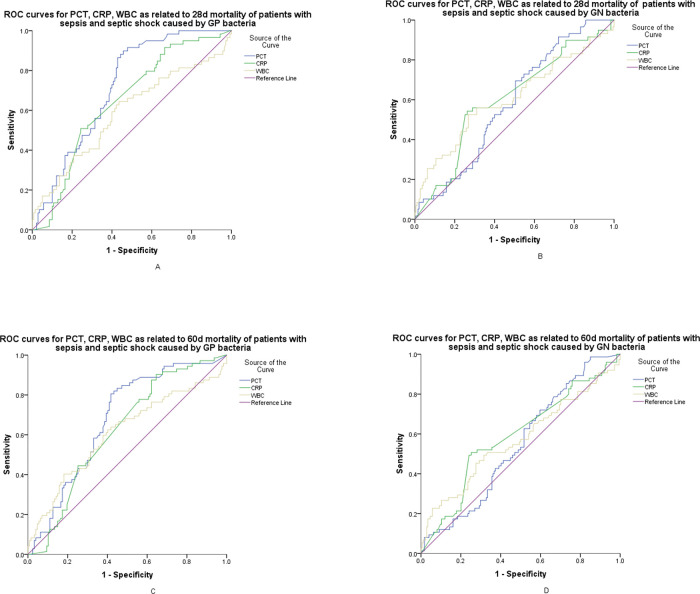
ROC curves for serum PCT, hs-CRP, and WBC. A: AUC demonstrates that serum PCT measures 0.718 (95% CI, 0.648-0.788), hs-CRP measures 0.614 (95% CI, 0.543-0.682), and WBC measures 0.588 (95% CI, 0.497-0.679) for the prognosis of early mortality of patients with sepsis and septic shock caused by GPB infections. B: AUC demonstrates that serum PCT measures 0.583 (95% CI, 0.507-0.659), hs-CRP measures 0.603 (95% CI, 0.520-0.686), and WBC measures 0.608 (95% CI, 0.518-0.697) for the prognosis of early mortality of patients with sepsis and septic shock caused by GNB infections. C: AUC demonstrates that serum PCT measures 0.678 (95% CI, 0.603-0.753), hs-CRP measures 0.600 (95% CI, 0.529-0.669), and WBC measures 0.587 (95% CI, 0.515-0.656) for the prognosis of late mortality in patients with sepsis and septic shock caused by GPB infections. D: AUC demonstrates that serum PCT measures 0.547 (95% CI, 0.473-0.621); hs-CRP, 0.588 (95% CI, 0.510-0.667), and WBC measures 0.570 (95% CI, 0.487-0.652) for the prognosis of late mortality of patients with sepsis and septic shock caused by GNB infections. ROC, receiver operating characteristic; PCT, procalcitonin; hs-CRP, high-sensitivity C-reactive protein; WBC, white blood cell; AUC, area under the curve; CI, confidence interval; GPB, gram-positive bacteria; GNB, gram-negative bacteria.

Figure 3-A,C show the ROC curves of PCT, hs-CRP, and WBC for early and late mortality prediction of patients with sepsis and septic shock caused by GPB. Pairwise comparisons of the AUC for early mortality were as follows: PCT *versus* hs-CRP, *p*=0.037; PCT *versus* WBC, *p*=0.021; and hs-CRP *versus* WBC, *p*=0.346. At a cut-off value of 0.73 ng/mL, PCT showed a sensitivity of 89.8%, a specificity of 54.3%, and an NPV of 92.7% with GPB infections. Pairwise comparisons of the AUC for 60-day mortality were as follows: PCT *versus* hs-CRP, *p*=0.096; PCT *versus* WBC, *p*=0.101; and hs-CRP *versus* WBC, *p*=0.817. At a cut-off value of 0.92 ng/mL, PCT showed a sensitivity of 80.6%, a specificity of 58.3%, and an NPV of 84.1% with GPB infections (shown in Table 4). Figure 3-B,D show the ROC curves of PCT, hs-CRP, and WBC for early and late mortality prediction of patients with sepsis and septic shock caused by GNB. Pairwise comparisons of the AUC for early mortality were as follows: PCT *versus* hs-CRP, *p*=0.690; PCT *versus* WBC, *p*=0.672; and hs-CRP *versus* WBC, *p*=0.944. For 60-day mortality, the values were as follows: PCT *versus* hs-CRP, *p*=0.382; PCT *versus* WBC, *p*=0.671; and hs-CRP *versus* WBC, *p*=0.751. Compared to GNB infections, the AUROC of early and late mortality prediction for PCT in GPB infections was higher (0.718 *versus* 0.583; 0.678 *versus* 0.547) (shown in Figure 3-A-D).

### The predictive values of CISs for early and late mortality with different gram stain bacteria infections

**Figure 4:**
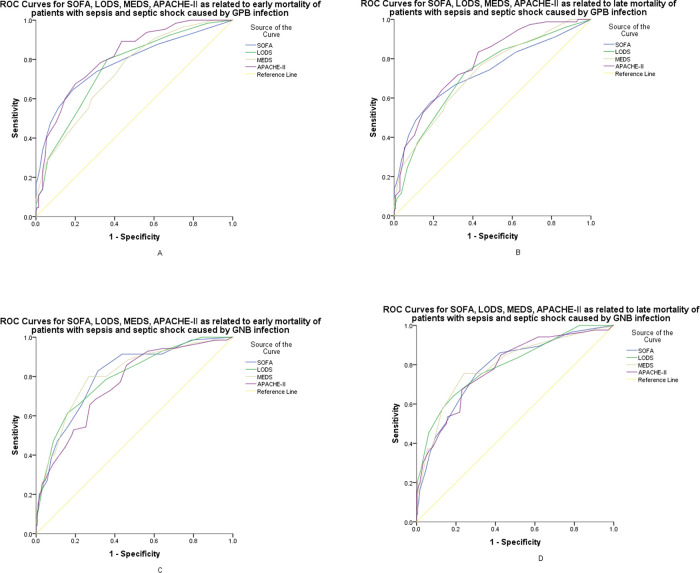
ROC curves for SOFA, LODS, MEDS, and APACHE-II. A: AUC demonstrates that SOFA measures 0.782 (95% CI, 0.709-0.854), LODS was 0.756 (95% CI, 0.687-0.826), 0.739 (95%CI, 0.669-0.808), and APACHE II measures 0.814 (95% CI, 0.754-0.874) for the prognosis of early mortality of patients with sepsis and septic shock caused by GPB infections. B: AUC demonstrates that SOFA measures 0.731 (95% CI, 0.657-0.805), LODS measures 0.723 (95% CI, 0.652-0.794), MEDS was 0.724 (95%CI, 0.654-0.794), and APACHE II measures 0.780 (95% CI, 0.718-0.843) for the prognosis of late mortality in patients with sepsis and septic shock caused by GPB infections. C: AUC demonstrates that SOFA measures 0.806 (95% CI: 0.747-0.864), LODS measures 0.796 (95% CI, 0.736-0.857), MEDS measures 0.807 (95%CI, 0.746-0.867), and APACHE II measures 0.761 (95% CI, 0.697-0.825) for the prognosis of early mortality in patients with sepsis and septic shock caused by GNB infections. D: AUC demonstrates that SOFA measures 0.782 (95% CI, 0.723-0.842), LODS was 0.795 (95% CI, 0.736-0.853), 0.796 (95% CI, 0.736-0.855), and APACHE II measures 0.783 (95% CI, 0.724-0.842) for the prognosis of late mortality in patients with sepsis and septic shock caused by GNB infections. ROC, receiver operating characteristic; PCT, procalcitonin; hs-CRP, high-sensitivity C-reactive protein; WBC, white blood cell; AUC, area under the curve; CI, confidence interval; GPB, gram-positive bacteria; GNB, gram-negative bacteria.

Figure 4-A,B show the ROC curves of SOFA, LODS, MEDS, and APACHE-II for early and late mortality prediction of patients with sepsis and septic shock caused by GPB. The optimal cut-off values of SOFA, LODS, MEDS and APACHE-II for early mortality prediction were 6.5, 3.5, 9.5, 18.5 points, respectively, with sensitivities of 64.6%, 80.0%, 80.0%, 67.7%, respectively, and specificities of 81.2%, 63.8%, 54.4%, 79.9%, respectively. For late mortality prediction, the sensitivities were 57.7%, 73.1%, 76.9%, 83.3%, and the specificities were 81.6%, 64.0%, 55.9%, 57.4%, at the cut-off values of 6.5, 3.5, 9.5, 14.5 points, respectively.

Figure 4-C,D show the ROC curves of SOFA, LODS, MEDS, and APACHE-II for early and late mortality prediction of patients with sepsis and septic shock caused by GNB. The optimal cutoff value of SOFA, LODS, MEDS and APACHE-II for early mortality prediction were 5.5, 5.5, 12.5, 18.5 points, respectively, with sensitivities of 82.9%, 61.4%, 80.0%, 65.7%, respectively, and specificities of 68.6%, 84.0%, 73.2%, 72.7%, respectively. For late mortality prediction, the sensitivities were 75.6%, 64.0%, 75.6%, 67.4% and the specificities were 69.7%, 81.5%, 75.8%, 77.0%, at the cut-off values of 5.5, 4.5, 12.5, 18.5 points, respectively.

## DISCUSSION

We evaluated four outcome prediction scoring systems and three biomarkers to predict early and late mortality among septis patients with BSIs. We found that CISs perform better than biomarkers for early and late mortality, especially for patients with GNB infection; the SOFA score offered better predictive performance than the MEDS score for early mortality. PCT achieved better predictive performance when used for GPB infections than hs-CRP and WBC for early mortality, probably performing better for GPB than for GNB.

The Third International Consensus Definitions for Sepsis and Septic Shock recommend that the SOFA score be used as a new clinical criterion for sepsis, and that the recommended criteria achieved moderate predictive performance for acute mortality [(In ICU patients with suspected infection: AUROC=0.74; 95% confidence interval [CI], 0.73-0.76); (for patients outside the ICU with suspected infection: AUROC=0.79; 95% CI, 0.78-0.80)] (1). This is comparable to the AUC of 80.5% in our study for early (28-day) mortality. Although the SOFA score uses physiological and laboratory variables alone and given that organ failure is an important predictor of the worst-case outcome, we found that the SOFA score performed better in predicting early mortality than the MEDS score. The MEDS score requires a subjective assessment of the likelihood of short-term mortality and may be less accurate in cases of higher illness severity. Jones et al. (25) found that the MEDS score had an AUC of 0.61 for patients admitted to the ED with septic shock. Similarly, Nguyen et al. (26) found MEDS to have an AUC of 0.63 in patients undergoing early goal-directed treatment in the ED. Our results for AUC were 0.761 for early mortality prediction and 0.744 for late mortality prediction, similar to the AUC of 0.78 found among patients with sepsis in the initial MEDS validation study (10). Patient selection may account for these different outcomes in that our study represents a more infirm group of patients with higher illness severity according to the sepsis-3 criteria.

The original APACHE score was developed in 1981 to classify groups of patients according to illness severity. It was divided into two sections: a physiology score to assess the degree of acute illness and a preadmission evaluation to determine the chronic health status of the patient (27). In 1985, the original model was revised and simplified to create APACHE-II (8), which is now the world’s most widely used severity-of-illness score. In APACHE-II, there are 12 physiological variables, with the worst value recorded during the first 24 h of a patient’s admission to the ICU used for each physiological variable. Furthermore, the effects of age and chronic health status are incorporated directly into the model and weighted according to their relative impact, likely rendering better predictive ability for late mortality. Our study found that the APACHE-II score had the largest AUC among the four prognostic scores and performed better than the MEDS score for late mortality prediction. This is in contrast to Pong JZ et al. (28), who found that the MEDS score outperformed the APACHE-II score in predicting 30-day in-hospital mortality among ED patients with suspected sepsis and fulfilled the systemic inflammatory response syndrome criteria. Chen YX et al. (29) found that the two scoring systems had similar AUC values for early mortality prediction among septic patients admitted to the ED. Patient selection may account for these different outcomes.

PCT, serving as a biomarker of bacterial infection, has been widely investigated for its prognostic value in patients with sepsis. A meta-analysis (30) including 23 studies with 3994 patients found that elevated PCT levels were associated with a higher risk for death. Nevertheless, a recently published study (31) also found that PCT was a promising biomarker for the prognosis of mortality in sepsis, with an AUROC of 0.81, which was higher than our results. This may be attributed to patient selection and PCT testing time. Given that PCT values were associated with bacterial types (23,32), we analyzed the predictive power of PCT for early and late mortality among sepsis patients with GNB and GPB infections, respectively; we found that AUCs were higher in GPB infections. This may be attributed to the fact that GPB lacks cell walls and stimulates PCT release through the production of distinct inflammatory cytokines alone (33). Consequently, to some extent, the PCT value may reflect the severity of the inflammatory response. Unlike GPB infections, GNB produces endotoxins that can also be released upon cell death, also affecting PCT levels (34).

We also found that PCT performed better than hs-CRP and WBC for the prediction of early mortality among sepsis patients with GPB infections; it did not show any advantage in mortality prediction before we segregated patients into two groups based on Gram stain testing. In contrast, the AUROC of CISs in early and late mortality prediction was larger for GNB infection than for GPB infection. To the best of our knowledge, this is the first study to demonstrate the predictive value of PCT in patients with GPB and GNB infections.

This study has some limitations. First, this is a subgroup analysis; although the subgroups have a large number of patients, it is still a post-hoc analysis of a single-center retrospective study. Therefore, the utility of these data is limited to emergency applications. Second, our data is just a snapshot of hospital admission, since sepsis severity is a continuum; a patient who is infected now may develop sepsis or septic shock tomorrow. Third, the predictive power of PCT might have been confounded by the fact that the intervals between the onset of symptoms and sampling were variable. Indeed, PCT levels can vary over the course of infection, especially during the first 6h of infection (35,36). Therefore, prospective multicenter studies using samples collected at consistent single or multiple time points, if possible, should be conducted for sepsis patients with BSIs in the ED to investigate whether real-life measurement of PCT contributes useful prognostic information, thereby improving the daily clinical management and outcomes of septic patients.

## CONCLUSIONS

CISs were more advantageous in the assessment of early and late prognosis, especially for GNB infections. However, for sepsis with GPB infection, PCT can be used to predict early mortality.

### Highlights of the Study

- There were significant differences in critical illness scores (CISs) and biomarkers between the death and survival groups in predicting early and late mortality in patients with sepsis and septic shock.
- CISs showed a significantly higher area under the curve (AUC) in the prediction of early and late mortality based on receiver operating characteristic curve analysis than biomarkers, especially for patients with gram-negative bacteria (GNB) infections.
- Procalcitonin (PCT) might perform better for gram-positive bacteria (GPB) infections than for GNB infections in the prognosis of patients with sepsis-3. Meanwhile, PCT performed better than high-sensitivity C-reactive protein and white blood cells with GPB infections.
- The Sequential Organ Failure Assessment score showed a higher AUC for predicting early mortality than the Mortality in Emergency Department Sepsis score.

## AUTHOR CONTRIBUTIONS

Yan ST participated in the study design and coordination, data acquisition and analysis, performed statistical analyses and interpretation, and drafted the manuscript. Zhang GQ conceived the study, participated in its design and coordination, and participated in data analysis and interpretation. All authors read and approved the final version of the manuscript.

## Figures and Tables

**Table 1:** Comparison of the clinical and biological characteristics of patients with sepsis and septic shock.

Characteristics	Sepsis N=666	Septic Shock N=168	*p*-value
Sex (n, %)			
Male sex	430 (64.6)	92 (54.8)	0.019
Age (years)	66.15 (64.32-67.98)	66.20 (62.80-69.59)	0.412
Site of infection (n,%)			
Respiratory infection	386 (58.0)	124 (73.8)	<0.001
Abdominal infection	159 (23.9)	27 (16.1)	0.030
Urinary infection	43 (6.5)	6 (3.6)	0.155
Catheter-related infection	52 (7.8)	8 (4.8)	0.172
Infective endocarditis	9 (1.4)	1 (0.6)	0.683
Others	17 (2.6)	4 (2.4)	1.000
Pathogens detected			
Monomicrobial			
Gram-positive bacteria	262 (39.3)	62 (36.9)	0.850
Gram-negative bacteria	314 (47.1)	79 (47.0)	0.641
Fungi	41 (6.2)	8 (4.8)	0.568
Polymicrobial	49 (7.4)	19 (11.3)	0.094
WBC (*10^9^/L)	10.81 (10.24-11.38)	12.55 (11.01-14.08)	0.042
PLT(*10^9^/L)	172.69 (162.16-183.23)	120.99 (102.00-139.97)	<0.001
HCT (%)	31.38 (30.70-32.05)	30.60 (29.02-32.18)	0.223
Na (mEq/L)	137.50 (136.88-138.11)	139.33 (137.95-140.71)	0.009
K (mg/dL)	4.14 (4.08-4.21)	4.07 (3.93-4.21)	0.443
CR (mg/dL)	146.13 (125.62-166.63)	154.40 (135.70-173.09)	0.062
TBIL (μmol/L)	33.54 (27.83-39.25)	36.77 (27.16-46.38)	0.010
hs-CRP (mg/L)	89.16 (84.15-94.16)	117.69 (107.87-127.51)	<0.001
PCT (ng/mL)	12.88 (9.89-15.86)	26.79 (17.89-35.69)	<0.001
SOFA	5.07 (4.80-5.34)	8.59 (7.86-9.32)	<0.001
MEDS	9.98 (9.67-10.28)	14.68 (14.17-15.19)	<0.001
LODS	3.23 (3.01-3.46)	5.90 (5.31-6.50)	<0.001
APACHE-II	15.43 (14.83-16.03)	21.69 (20.23-23.14)	<0.001
Early mortality (n, %)	114 (17.1)	100 (59.8)	<0.001
Late mortality (n, %)	162 (24.3)	111 (66.1)	<0.001

WBC, white blood cell; PLT, platelet; HCT, hematocrit; CR, creatinine; TBIL, total bilirubin; hs-CRP, high-sensitivity C-reactive protein; PCT, procalcitonin; SOFA, Sequential organ failure assessment; LODS, Logistic organ dysfunction system; MEDS, Mortality in emergency department sepsis; APACHE-II, Acute Physiology and Chronic Health Evaluation II.

**Table 2:** Prognostic scores and laboratory findings for sepsis and septic shock predicting early (A) and late (B) mortality.

Biomarkers and Prognostic Scores	Death n=214	Survival n=620	*p*-value
A			
PCT (ng/mL)	3.03 (0.87-16.09)	1.42 (0.25-10.66)	0.001
hs-CRP (mg/L)	117.69 (89.16-130.00)	89.16 (47.68-110.00)	<0.001
WBC (×10^9^/L)	12.42 (6.98-17.35)	9.43 (6.62-13.32)	<0.001
SOFA score	8.0 (6.0-11.0)	4.0 (3.0-6.0)	<0.001
MEDS score	14.0 (11.0-16.0)	10.0 (8.0-13.0)	<0.001
LODS score	6.0 (4.0-7.0)	3.0 (1.0-4.0)	<0.001
APACHE-II score	21.0 (16.0-26.0)	14.0 (10.0-18.0)	<0.001

Biomarkers and Prognostic Scores	Death n=273	Survival n=561	*p*-value
B			
PCT (ng/mL)	2.75 (0.69-13.24)	1.44 (0.24-12.76)	0.007
hs-CRP (mg/L)	100.93 (89.16-126.50)	89.16 (47.84-110.10)	<0.001
WBC (×10^9^/L)	11.98 (6.97-16.98)	9.27 (6.63-13.29)	0.001
SOFA score	7.0 (5.0-10.0)	4.0 (3.0-6.0)	<0.001
MEDS score	13.0 (11.0-16.0)	9.0 (8.0-12.0)	<0.001
LODS score	5.0 (3.0-7.0)	3.0 (1.0-4.0)	<0.001
APACHE-II score	20.0 (16.0-25.0)	13.0 (10.0-17.5)	<0.001

Median value with interquartile range; statistical analysis was performed using the Mann-Whitney U test; A: prediction of early mortality; B: prediction of late mortality. PCT, procalcitonin; hs-CRP, high-sensitivity C-reactive protein; WBC, white blood cell; SOFA, Sequential organ failure assessment; LODS, Logistic organ dysfunction system; MEDS, Mortality in Emergency Department Sepsis; APACHE-II, Acute Physiology and Chronic Health Evaluation II.

**Table 3:** Diagnostic values of prognostic scores and laboratory findings in predicting early (A) and late (B) mortality among patients with sepsis and septic shock.

Biomarkers and Prognostic Scores	AUC	Best Cut-off	Sensitivity (%)	Specificity (%)	PPV (%)	NPV (%)
A						
PCT	0.634	0.84	84.8	43.3	36.3	87.8
hs-CRP	0.640	110.92	55.9	74.9	46.7	81.8
WBC	0.595	11.51	55.9	66.8	39.1	79.8
SOFA	0.805	5	88.5	56.5	44.0	92.7
MEDS	0.761	13	64.9	73.4	48.5	84.4
LODS	0.785	3	88.5	45.1	38.3	91.1
APACHE-II	0.778	15	85.8	54.2	41.9	90.8

Biomarkers and Prognostic Scores	AUC	Best Cut-off	Sensitivity (%)	Specificity (%)	PPV (%)	NPV (%)
B						
PCT	0.596	0.60	83.2	38.8	42.2	80.6
hs-CRP	0.605	110.915	48.9	74.7	51.7	73.3
WBC	0.586	11.365	53.8	66.2	46.3	72.6
SOFA	0.754	5	79.7	56.8	50.0	83.8
MEDS	0.744	13	60.4	75.4	57.1	77.8
LODS	0.755	3	83.4	46.1	45.6	83.7
APACHE-II	0.774	15	83.4	57.4	51.5	86.5

AUC, area under the curve; PPV, positive predictive value; NPV, negative predictive value; PCT, procalcitonin; hs-CRP, high-sensitivity C-reactive protein; WBC, white blood cell; SOFA, Sequential organ failure assessment; LODS, Logistic organ dysfunction system; MEDS, Mortality in emergency department sepsis; APACHE-II, Acute Physiology and Chronic Health Evaluation II.

**Table 4:** Diagnostic values of laboratory findings in predicting early and late mortality of patients with sepsis and septic shock caused by GPB or GNB.

Subgroup characteristics	Biomarkers	AUC	Cut-off	Sensitivity (%)	Specificity (%)	PPV (%)	NPV (%)
Early mortality of patients with Sepsis and septic shock caused by GP bacteria	PCT	0.718	0.73	89.8	54.3	45.3	92.7
	hs-CRP	0.646	110.73	50.8	75.7	46.2	78.7
	WBC	0.588	10.93	64.4	56.4	38.4	79.0
Early mortality of patients with Sepsis and septic shock caused by GN bacteria	PCT	0.583	2.85	69.5	49.5	30.0	83.2
	hs-CRP	0.603	113.28	54.2	73.7	40.5	84.0
	WBC	0.608	12.40	52.5	73.2	37.8	83.2
Late mortality of patients with Sepsis and septic shock caused by GP bacteria	PCT	0.678	0.92	80.6	58.3	51.3	84.1
	hs-CRP	0.622	48.28	90.3	35.4	43.6	86.5
	WBC	0.609	11.08	62.5	59.8	46.9	92.7
Late mortality of patients with Sepsis and septic shock caused by GN bacteria	PCT	0.547	2.85	62.7	48.3	34.3	74.3
	hs-CRP	0.588	113.28	50.7	74.7	47.6	78.1
	WBC	0.570	11.51	49.3	68.4	40.2	75.8

GPB, gram-positive bacteria; GNB, gram-negative bacteria; AUC, area under the curve; PPV, positive predictive value; NPV, negative predictive value; PCT, procalcitonin; hs-CRP, high-sensitivity C-reactive protein; WBC, white blood cell.

## APPENDIX

**Supplemental File 1:** MEDS score with neutrophil bands >5% not included as these were not reported by the laboratory, giving a maximum total score of 24 if all the variables are present.^a^

Clinical variables	Weighted scores
Terminal illness^b^	6
Age >65 years	3
Tachypnea^c^ or hypoxia^d^	3
Shock	3
Platelets <150×10^9^/L	3
Altered mental state	2
Nursing home resident	2
Lower respiratory infection	2
Sum	24

Abbreviations: MEDS, Mortality in emergency department sepsis.

a Adapted from Vorwerk C et al. (9)

b Terminal illness is defined as metastatic cancer or a disease condition with a >50% likelihood of predicted fatality within 30 days.

c Respiratory rate >20 per minute.

d Pulse oximetry <90% or need for oxygen supplementation of 0.4 FiO_2_ or higher to maintain adequate oxygenation.

**Supplemental File 2:** Criteria of the SOFA score.^a^

System variables	0	1	2	3	4
Respiration					
PaO_2_/FiO_2_,mmHg (kPa)	≥400 (53.3)	<400 (53.3)	<300 (40)	<200 (26.7) with respiratory support	<100 (13.3) with respiratory support
Coagulation					
Platelets, ×10^3^/µL	≥150	<150	<100	<50	<20
Liver					
Bilirubin, mg/dL (µmol/L)	<1.2 (20)	1.2–1.9 (20–32)	2.0–5.9 (33–101)	6.0–11.9 (102–204)	≥12.0 (204)
Cardiovascular	MAP ≥70 mmHg	MAP <70 mmHg	Dopamine <5 or Dopamine 5.1–15 or Dopamine >15 or Dobutamine	epinephrine <0.1 or epinephrine >0.1 or b norepinephrine >0.1^b^	norepinephrine >0.1^b^
Central nervous system					
Glasgow Coma Scale Score^c^	15	13-14	10-12	6-9	<6
Renal					
Creatinine, mg/dL (µmol/L)	<1.2 (110)	1.2–1.9 (110–170)	2.0–3.4 (171–299)	3.5–4.9 (300–440)	>5.0 (440)
Urine output, mL/d				<500	<200

Abbreviations: SOFA, Sequential organ failure assessment; FiO_2_, fraction of inspired oxygen; MAP, mean arterial pressure; PaO_2_, partial pressure of oxygen

a Adapted from Vincent et al. (12)

b Catecholamine doses are given as µg/kg/min for at least 1h.

c Glasgow Coma Scale scores range from to 3–15; higher scores indicate better neurological function.

**Supplemental File 3:** Criteria of the LODS score..^a^

Organ system	Parameter	5	3	1	0	1	3	5
Neurologic	GCS	3–5	6–8	9–13	14–15			
Cardiogenic	HR (beats/min)	<30			30–139	≥140		
		or			and	or		
	SBP (mmHg)	<40	40–69	70–89	90–239	240–269	≥270	
Renal	Urea nitrogen (mmol/L)				<6	6–9.99	10–19.9	≥20
	(g/L)				<0.36	0.36–0.59	0.60–1.19	≥1.2
					and	or	or	
	Creatinine (μmol/L)				<106	106–140	≥141	
	(mg/dL)				<1.20	1.20–1.59	≥1.6	
					and		or	
	Urine output (L)	<0.5	0.5–0.74		0.75–9.99		≥10	
Pulmonary	PaO_2_/FiO_2_ (mmHg)		<150	≥150	no MV			
	(on MV or CPAP)				no CPAP			
	PaO_2_/FiO_2_ (kPa)		<19.9	≥19.9	no IPAP			
Hematologic	Leukocytes (×10^9^/L)		<1.0	1.0–2.4	2.5–49.9	≥50		
				or	and			
	Platelets (10^9^/L)			<50	≥50			
Hepatic	Bilirubin, (μmol/L)				<34.2	≥34.2		
	(mg/dL)				<2.0	≥2.0		
					and	or		
	PT time (s) (above normal)				≤3	>3		
	above standard (%)			<25	≥25			

Abbreviations: LODS, logistic organ dysfunction system; GCS, Glasgow coma scale; HR, heart rate; SBP, systolic blood pressure; PaO_2_, partial pressure of oxygen; FiO_2_, fraction of inspired oxygen; MV, machine ventilation; CPAP, continuous positive airway pressure; IPAP, intermittent positive airway pressure; PT, prothrombin time.

a Adapted from Le Gall JR, et al. (15)
